# Genome-wide analysis of Dof transcription factors reveals functional characteristics during development and response to biotic stresses in pepper

**DOI:** 10.1038/srep33332

**Published:** 2016-09-22

**Authors:** Won-Hee Kang, Seungill Kim, Hyun-Ah Lee, Doil Choi, Seon-In Yeom

**Affiliations:** 1 Department of Agricultural Plant Science, Institute of Agriculture & Life Science, Gyeongsang National University, 660-701, South Korea; 2 Department of Plant Science, Plant Genomics and Breeding Institute, College of Agriculture and Life Sciences, Seoul National University, Seoul 151-921, South Korea

## Abstract

The DNA-binding with one zinc finger proteins (Dofs) are a plant-specific family of transcription factors. The Dofs are involved in a variety of biological processes such as phytohormone production, seed development, and environmental adaptation. Dofs have been previously identified in several plants, but not in pepper. We identified 33 putative *Dof* genes in pepper (*CaDofs*). To gain an overview of the *CaDof*s, we analyzed phylogenetic relationships, protein motifs, and evolutionary history. We divided the 33 *CaDofs*, containing 25 motifs, into four major groups distributed on eight chromosomes. We discovered an expansion of the *CaDof*s dated to a recent duplication event. Segmental duplication that occurred before the speciation of the Solanaceae lineages was predominant among the *CaDof*s. The global gene-expression profiling of the *CaDof*s by RNA-seq analysis showed distinct temporal and pathogen-specific variation during development and response to biotic stresses (two TMV strains, PepMoV, and *Phytophthora capsici*), suggesting functional diversity among the *CaDof*s. These results will provide the useful clues into the responses of *Dofs* in biotic stresses and promote a better understanding of their multiple function in pepper and other species.

Plants constantly encounter biotic and abiotic stresses. Biotic stresses cause serious reduction in crop production and quality. To overcome biotic stresses, plants have various strategies involving the perception of pathogen attacks and the regulation of signaling networks^1^,^2^,^3^. Transcription factors (TFs) are vital proteins that control cellular processes by regulating the expression of downstream target genes through the recognition of specific DNA sequence elements in promoter regions^4^. TFs also play roles in plant innate immunity^5^. Multitudinous TF families exist in plants, and more than 60 TF families have been identified by bioinformatics analysis and manual study of plant genomes^6^. Several plant-specific TF families have been identified, including the DNA-binding with one zinc finger proteins (Dofs).

The Dofs play key roles in many biological process such as seed germination, dormancy, photosynthesis, flowering, light-regulated gene expression, guard cell-specific gene expression, and stress response^4^,^7^,^8^. The Dofs generally have 200–400 amino acids with a highly conserved 52 amino-acid Dof domain at the N-terminal end and a variable domain for transcriptional regulation at the C-terminal end. The Dof domain, is a DNA-binding domain that contains a C2C2-type zinc finger motif that recognizes a cis-regulatory element with a common core sequence of 5′-(A/T)AAAG -3′, except a pumpkin Dof protein designated as AOBP that recognizes an 5′-AGTA-3′ sequence^4^,^7^,^8^. The Dof domain is bifunctional, showing both DNA-binding and protein-protein interaction activities^8^,^9^,^10^,^11^,^12^. The variable domain at the Dof C-terminus might be involved in the diverse functions of distinct Dofs via interactions with different regulatory proteins and gene promoters^13^,^14^.

Since the first Dof was identified in maize^15^, many putative Dof genes (*Dof*s) have been reported in a variety of plant species, such as the 36 *Dof*s in Arabidopsis^16^, 30 *Dof*s in rice^16^, 41 *Dof*s in poplar^17^, 31 *Dof*s in wheat^18^, 28 *Dof*s in sorghum^19^, 78 *Dof*s in soybean^20^, 34 *Dof*s in tomato^21^, and 76 *Dof*s in Chinese cabbage^22^. Along with the rapid expansion and completion of genomic sequencing, more *Dof*s in various plants will be identified and could promote a better understanding of *Dofs* function.

Much research has suggested that Dofs have multiple roles such as plant-specific physiological process, growth, development, and photosynthesis. In maize, Dof1 and 2 are involved in gene expression for carbon metabolism^23^. ZmDof1/MNB1a regulates the PEPC gene in association with light response and nitrogen assimilation^24^. In Arabidopsis, DAG1 and DAG2 are involved in seed germination^25^,^26^, and CDF1, 2, 3, and 5 are associated with the photoperiodic control of flowering^27^,^28^. COG1 and OBP3 regulate phytochrome signaling^29^,^30^. In rice, OsDof3, OsDof12, and RPBF are involved in gibberellin-regulated gene expression, the photoperiodic control of flowering, and the regulation of seed-storage protein, respectively^9^,^31^,^32^,^33^. In potatoes, StDof1 regulates the KST1 gene in association with guard-cell specificity^34^. In soybean, GmDof4 and GmDof11 are associated with lipid biosynthesis^35^. Some *Dof*s are involved in responses to abiotic and biotic stresses. SlCDF1 and SlCDF3 in tomato regulate the COR15, RD29A, and RD10 genes in association with drought and salt tolerances^36^. TaDof14 and TaDof15 in wheat were significantly up-regulated under drought conditions^18^. But, reports of Dof family involvement in responses biotic stress have been very limited.

Pepper, a member of Solanaceae plants such as tomato, potato and tobacco, comprise large portion of crops in the world as major vegetable for most global cuisines with pungency, and are exposed to various devastating diseases by *Tobacco mosaic virus* (TMV), *Pepper mottle virus* (PepMov), *Tomato spotted wilt virus* (TSWV), *Phytophthora capsici, Collectricum spp.* and so on^37^,^38^,^39^. These biotic stresses cause serious reduction in yield and quality. These days, several efforts to clone resistance genes or candidates were reported to overcome pathogen attacks in pepper, but the defense-response mechanism and network still remain unknown. Understanding diverse roles of Dof family in plant biological process could be feasible approach to dissect defense response in biotic stresses and improve crop disease resistance or tolerance. However, in pepper, the function of the Dof family is still largely unknown. To get a comprehensive understanding of Dofs in pepper, a genome-wide identification and expression analysis of the Dof family was performed in the present study. Subsequently, we performed a detailed analysis of gene structures, conserved motifs, duplication status, chromosomal distribution, and phylogenic relationships to explore the evolutionary history of *Dof* expansion in pepper. To determine the probable functions of the pepper *Dof*s (*CaDof*s), we evaluated expression profiles under three pathogen challenges [*Tobacco mosaic virus* (TMV), *Pepper mottle virus* (PepMoV), and *Phytophthora capsici*] to understand the response of *CaDof*s to biotic stresses using RNA-seq data. The results will provide novel insights into the responses of *CaDof*s against pathogens and could promote a better understanding *Dof* function in other species.

## Results

### Identification of the *Dof* gene family in pepper

We identified 33 putative *CaDof*s from the *Capsicum annuum* ‘CM334’ (hereafter ‘CM334’) genome. Additionally, we obtained 21 putative *CaDof*s from the Plant Transcription Factor Database (Plant TFDB). We aligned the 33 putative *CaDof*s from the ‘CM334’ genome with 21 corresponding genes from the Plant TFDB. All of the candidate *CaDof*s from the Plant TFDB were matched to corresponding genes among the 33 putative *CaDof*s. Therefore, we used only the 33 *CaDof*s from *C. annuum* ‘CM334’ for further investigation. To verify the sequences of the *CaDof*s, we aligned all of the *CaDof*s with corresponding transcripts or genes from the ‘CM334’ RNA-seq^40^, pepper EST^41^, and *C. annuum* ‘Zunla-1’ CDS^42^ databases and manually corrected. We designated the generated *CaDof*s as *CaDof*01–33 according to their locations on the chromosomes. The number of *Dof*s in the pepper genome was similar to those in Arabidopsis (36 *Dof*s), rice (30 *Dof*s) and tomato (34 *Dof*s)^16^,^21^. All of the *CaDof*s contained the highly conserved Dof domain including the zinc finger motif (Supplementary Fig. S1). The conserved Dof domain consists of 52 basic residues located in the N-terminal region. Twenty-six of the 52 residues were perfectly conserved (100% conserved) in all 33 CaDofs. The Dof domains were also highly conserved among the Dofs of other plants, such as Arabidopsis, rice, and tomato (Supplementary Fig. S2). Information about the *CaDof*s; such as the gene ID, position on the chromosomes, number of nucleotides and amino acids, isoelectric point (pI), and molecular weight (MW); is presented in Table 1. The size of the coding sequence (CDS) of the *CaDof*s ranged from 525 bp to 1,512 bp, with deduced polypeptide sizes ranging from 173 amino acids to 503 amino acids. The pIs of the deduced CaDofs ranged from 3.61 to 9.79, and the MWs ranged from 15.8 kD to 54.27 kD.

### Phylogenetic relationships of the *Dof* family in pepper and tomato plants

**Figure 1:**
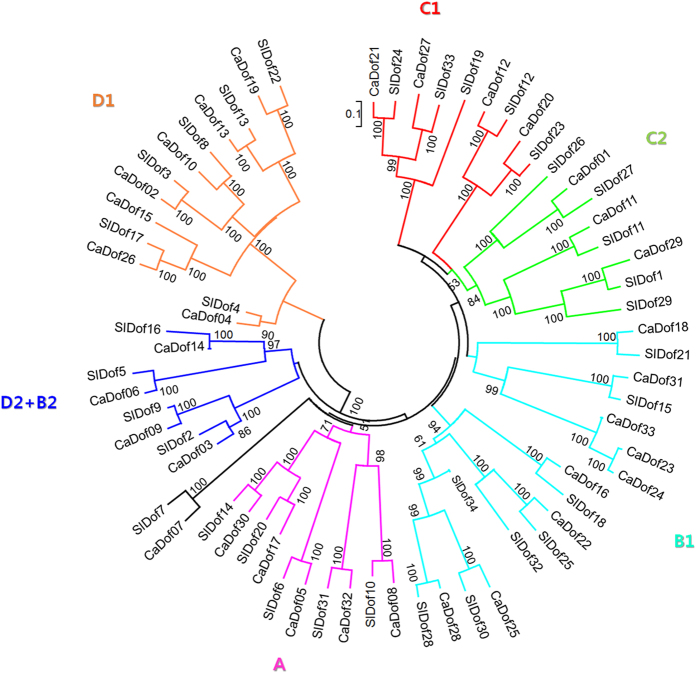
Phylogenetic tree of pepper and tomato *Dof* genes. The full length amino acid sequences of CaDofs and SlDofs were aligned by ClustalW. The Neighbor-Joining phylogenetic tree with 1,000 bootstrap replicates was constructed using 33 pepper Dof proteins and 34 tomato Dof proteins. Each specific color represents each Dof subclass.

To examine the phylogenetic relationships between the *Dof* families in pepper and tomato, we performed multiple sequence alignment of the 33 *CaDof*s and 34 tomato *Dof*s (*SlDof*s) using ClustalW. We constructed an unrooted phylogenetic tree using the neighbor-joining method with alignment of the full-length Dofs from tomato and pepper. Based on the resulting phylogenetic tree, we categorized the *CaDof*s and *SlDof*s into four major groups (A, B, C, and D) and seven subgroups (Fig. 1 and Supplementary Fig. S3). We named the pepper groups based on the corresponding groups in tomato^21^. Groups B and C were the largest major groups, each comprising 17 genes (accounting for 25.3%) in pepper and tomato. Groups B and C were divided into two subgroups: B1 and B2 and C1 and C2, respectively. Subgroup B1, which consisted of nine *CaDof*s and eight *SlDof*s, was the largest subgroup in both pepper and tomato. Group A and subgroups C1, C2, and D1 contained 10, 9, 8, and 13 members, respectively, from the two plants. Subgroups B2 and D2 were not clearly separated and clustered into the same subgroup with four *CaDof*s and four *SlDof*s. Most of the *CaDof*s were clustered with *SlDof*s, and each subgroup had a high bootstrap value, suggesting that the *CaDof*s have a close phylogenetic relationship with that of the *SlDof*s.

### Identification of conserved motifs and analysis of gene structures among the CaDofs

**Figure 2:**
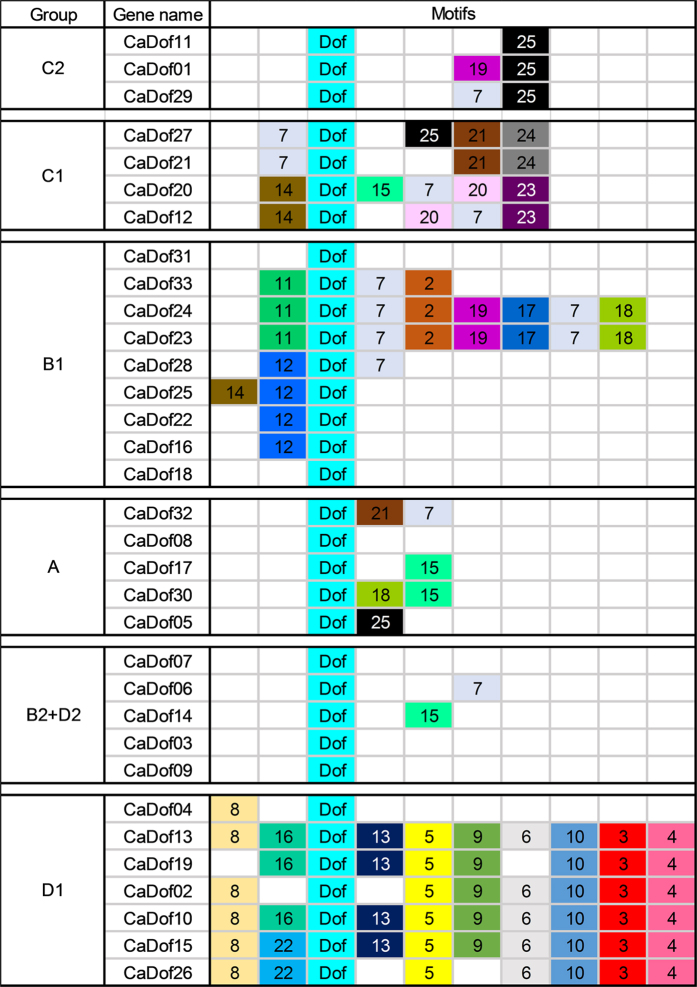
Schematic diagram of the conserved motifs in pepper 33 *Dof* genes. Motifs were identified by MEME program. Each number with colored box means each motif. Light blue boxes labeled Dof represent motif 1. The relative position of each motif in all Dof proteins is shown. Consensus sequence of the defined motifs by MEME are listed in Table S1.

To identify the diversity and conservation among the *CaDof*s, we investigated the conserved motifs using the MEME suite (http://meme-suite.org/tools/meme). A total of 25 distinct motifs were predicted among all 33 CaDofs (Fig. 2). The size of the identified motifs ranged from 8 amino acids to 113 amino acids. The consensus sequences of the motifs are presented in Supplementary Table S1. The motifs of closely related genes within each group in the phylogenetic tree shared common sequences and positions (Fig. 2). Most of the subgroups included several specific motifs in the C-terminal regions. Motif 1 was uniformly found in all 33 *CaDof*s and represented the pepper Dof domain (Fig. 2 and Supplementary Table S1). Group A and subgroups B2 and D2 represent the simplest composition of motifs, with no specific motifs except for the Dof domain. Although subgroup B1 had several motifs (motifs 2, 7, 17, 18, and 19) at the C-terminal regions, it appeared to be characterized by motifs 11 and 12 at the N-terminal region. One *CaDof* (*CaDof*31) in subgroup B1 did not contain motifs 11 and 12. Subgroup C1 contained four specific motifs on both sides of the Dof domain: motifs 7 and 14 in the N-terminal region and motifs 24 and 23 in the C-terminal region. Subgroup C2 contained one specific motif (motif 25). Subgroup D1, which showed the most diverse composition of motifs among the subgroups, was characterized by five motifs (motifs 3, 4, 5, 8, and 10). Motif 8 was located in the N-terminal region in all of the D1 members except for *CaDof*19. Conserved motifs 3, 4, 5, and 10 at the C-terminus were detected in seven of the eight *Dof*s in subgroup D1. *CaDof*04 in subgroup D1 contained only two motifs (motif 8 and the Dof domain), possibly because it had a shorter gene sequence than the other D1 members.

**Figure 3:**
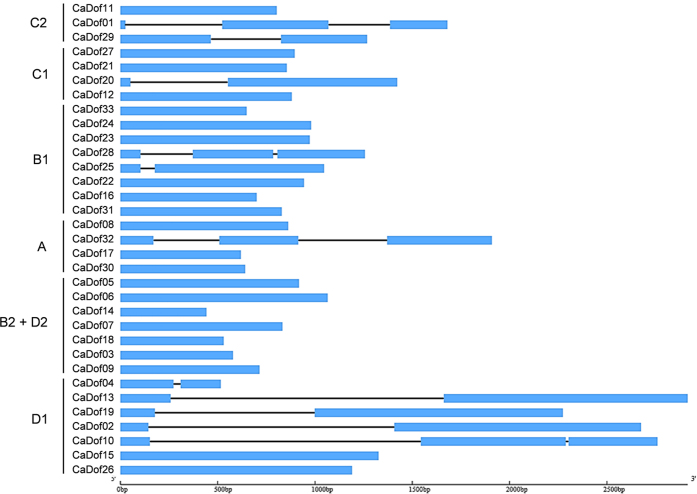
Exon and intron structure of *Dof* genes from pepper. Blue boxes and black lines represent exons and introns, respectively. The scale bar on the bottom line indicates size of genes.

In order to obtain further insight into the structural diversity of the *CaDof*s, we analyzed the exon-intron organization. As shown in Fig. 3, 22 of the *CaDof*s had no introns, whereas seven and four *CaDof*s had one and two introns, respectively. Those exon-intron structures were similar to those of the *Dof*s in Arabidopsis, rice, and tomato^16^,^21^. Most of the introns were generally located upstream of the Dof domain; only three genes (*CaDof*04, 29, and32) contained introns downstream of the Dof domain. Most of the genes within a given group showed similar organizations of exons and introns. Most of the genes from subgroup D1 contained one intron, and the intron sizes and positions were comparable among the genes in D1. Subgroups B2 and D2 contained no introns. The structures of the *CaDof*s in group A and subgroups B1, C1, and C2 were more variable than those in subgroups D1, B2, and D2. The distribution of motifs together with the gene structure within each group indicated the phylogenetic relationships among the *CaDof*s. Those results indicate that the conservation and specificity of gene characters among the subgroups could be related to functional diversity among the Dofs.

### Chromosomal location and gene duplication of *CaDof*s

**Figure 4:**
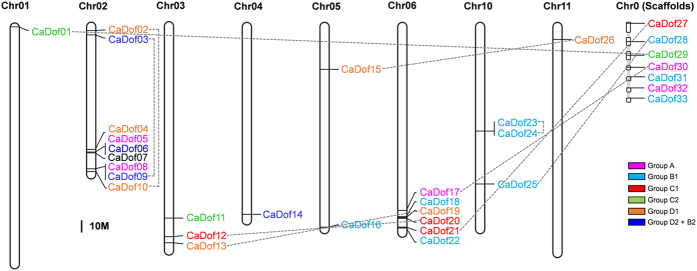
Chromosomal locations and duplication events of *CaDof* genes in pepper. The locations of *CaDof*s were based on physical locations. The four chromosomes without *CaDof*s (chromosome 7, 8, 9, and 12) were excluded in this figure. The numbers on the top indicate each chromosome number. The chromosome 0 (Chr0) means seven different scaffolds containing unassigned *CaDof*s to any of the 12 pepper chromosomes. Dotted lines indicate 10 pairs of paralogous gene of duplication events. *CaDof*s have been colored according to their groups of phylogenetic tree. Scale bar represents a 10 Mb distance of chromosome.

To determine the genomic localization of the *CaDof*s, we aligned each *CaDof* as query sequence against the pepper genome database using BLAST. The positions of the *CaDof*s on the chromosomes had an uneven distribution. Twenty-six genes were located on 8 of the 12 chromosomes, with no genes mapping to chromosomes 7, 8, 9, and 12 (Fig. 4). Nine *CaDof*s were mapped to chromosome 2. Among them, *CaDof*05, 06, and 07 and *CaDof*08 and 09 were clustered together, respectively. Chromosome 6 included six *CaDof*s, which were clustered in two groups (*CaDof*18, 19, and 20 and *CaDof*21 and 22, respectively) on the end of the long arm. Chromosomes 3 and 10 each contained three genes, and *CaDof*23 and 24 were clustered together. Chromosome 5 contained two genes. Chromosomes 1, 4 and 11 each contained only one gene. Seven of the 33 *CaDof*s (*CaDof*27–33) were assigned to chromosome 0 that means unassigned pepper sequences to any of the 12 pepper chromosomes.

To detect potential duplications of the *CaDof*s, we identified 10 putative pairs of *CaDof* paralogs based on the chromosomal localization and the neighbor-joining tree (Figs 1 and 4). Each pair of paralogs shared high sequence similarity. The identified paralogs are connected by the lines shown in Fig. 4. Seven of the paralog pairs were putative segmental duplication events, and three pairs were distributed at the same chromosome level. Only one putative pair (*CaDof*23 and 24) was possibly the result of tandem duplication. Overall, those results suggest that segmental duplication predominated in the expansion of the *CaDof* family in peppers, although tandem duplication was also involved.

In order to estimate the dates of the duplication events, we estimated the nonsynonymous substitution rate (Ka), the synonymous substitution rate (Ks), and the Ka/Ks ratio. The approximate dates of the duplication events are presented in Table 2. The nine segmental duplications (Fig. 4) of the *CaDof*s took place from 194.48 million years ago (Mya) to 37.06 Mya, and one tandem duplication (Fig. 4) occurred approximately 1.45 Mya. With the speciation data between pepper and tomato around 19.2 Mya^30^, all the segmental duplications occurred before the pepper/tomato split, which is also supported by the phylogenic tree shown in Fig. 1. The Ka/Ks ratios of five segmental duplication pairs and the tandem duplication pair were >0.3, suggesting that significant functional divergence among those *CaDof*s might have occurred after the duplication events. The Ka/Ks ratios of all the duplicate pairs were <1.0, indicating that the *CaDof*s evolved under negative selection acting against protein-coding changes.

### Expression patterns of *CaDof*s in various developmental stages

**Figure 5:**
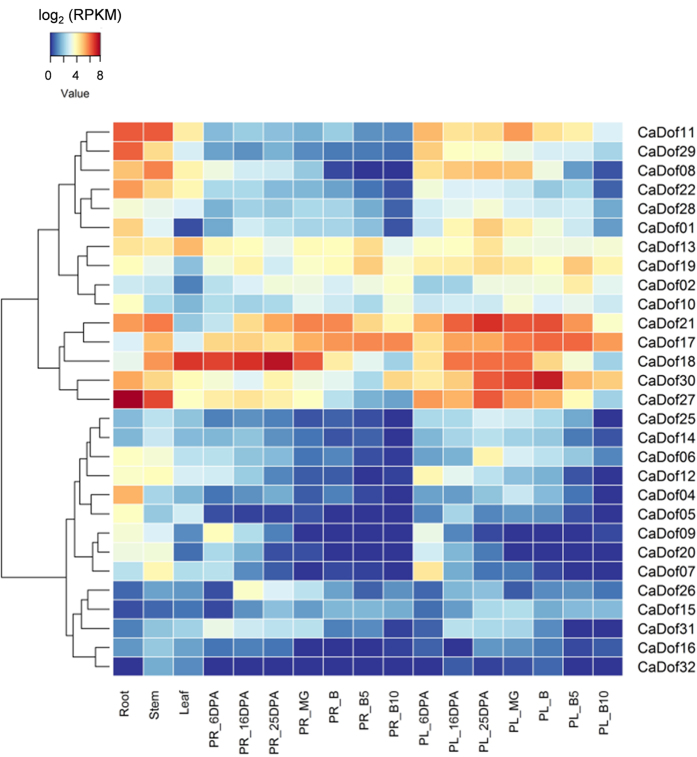
Tissue specific expression analysis of *CaDof*s. The heat map was constructed by RNA-seq from *C. annuum* ‘CM334’. The 29 *CaDof* genes were used to construct heat map. The 4 *CaDof* genes (*CaDof*03, 23, 24, and 33), which are absent data, were excluded. Blue and red colors represent relatively low and high expression (log_2_ RPKM value), respectively. PR, pericarp; PL, placenta; MG, mature green stage; B, breaker stage; DPA, days post-anthesis; B5 and 10, 5 and 10 days post-breaker.

To investigate the involvement of the *CaDof*s in pepper growth and development, we generated a heat map of the global transcription patterns of the *CaDofs* based on publicly available RNA-seq data for five tissues (root, stem, leaf, pericarp, and placenta), including seven developmental stages of the pericarp and placenta (Fig. 5). The expression profiles of each *CaDof* revealed various patterns in different organs and stages. *CaDof*13, 17, 18, 19, 21, 27, and 30 were expressed at relatively high levels, while *CaDof*14, 15, 16, 25, 31, and 32 were expressed at relatively low levels in all the tested tissues. The expression levels of *CaDof*11, 29, and 08 were high in the root and stem and higher in the placenta from 6 days post anthesis (PL_6DPA) to the mature green (MG) stage compared with those in the placenta at other stages, but they were low in the pericarp at all stages. *CaDof*22 was expressed at higher levels in the root and stem than in the other tissues. Although *CaDof*21 was expressed at relatively high levels in all the tested tissues, the expression levels in the placenta at several stages [PL_16 and 25DPA, MG, and breaker (B)] were higher than those in the other tissues. In comparison, *CaDof*30 was also highly expressed in the placenta, especially at the B stage. *CaDof*18 was constitutively expressed in most of the tested tissues, with especially high expression in some PR stages (PR 6, 16, and 25DPA and MG). *CaDof*27 showed the highest expression level in the roots among all the *CaDof*s. *CaDof*04 was expressed at a high level only in roots and was undetectable in the other organs. Taken together, those results indicate that the *CaDof*s are possibly involved in biological functions during plant development.

### Responses of *CaDof*s to TMV, PepMoV, and *P. capsici* pathogen challenges

‘CM334’ is well known as a powerful resource for the development of resistant lines against several viruses and *P. capsici*^37^,^38^,^39^. To examine whether *CaDof*s are involved in responses to biotic stresses in pepper, we analyzed the expression profiles of the *CaDof*s using RNA-seq data from ‘CM334’ challenged with TMV Pathotype 0 (TMV-P0), TMV-P2, PepMoV, and *P. capsici* (Supplementary Table S2). ‘CM334’ is resistant to TMV-P0, PepMoV, and *P. capsici* but susceptible to TMV-P2. We inoculated ‘CM334’ leaves with TMV-P0, TMV-P2, PepMoV, and *P. capsici* and then sampled at 0, 1, 2, and 3 days post inoculation. We used the leaf samples to construct RNA-seq libraries, which we subsequently sequenced. We then generated heat maps of the transcript profiles of the *CaDof*s with each gene expression value normalized by each control sample.

**Figure 6:**
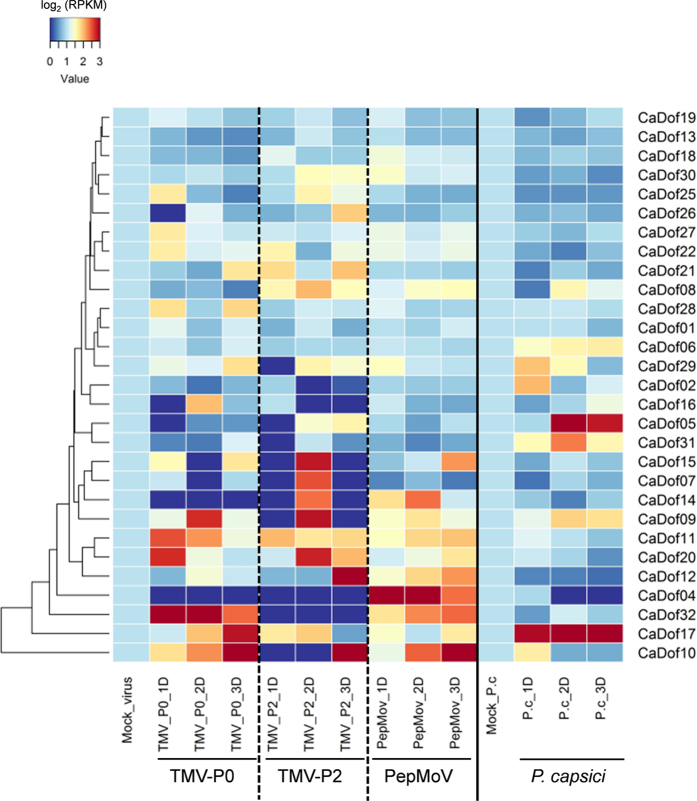
Biotic stress specific expression analysis of *CaDof*s. The heat map was constructed by RNA-seq from three viruses (TMV-P0, TMV-P2 and PepMoV) and *Phytophthora capsici* inoculated *C. annuum* ‘CM334’. ‘CM334’ is resistant to TMV-P0, PepMoV, and *P. capsici* but susceptible to TMV-P2.The 29 *CaDof* genes were used to construct heat map. The 4 *CaDof* genes (*CaDof*03, 23, 24, and 33), which are absent data, were excluded. For expression pattern after virus inoculation, mean of RPKM value in triplicate data sets were used. This data was normalized by control samples (Mock_virus and Mock_P.c). Blue and red colors represent relatively low and high expression (log_2_ RPKM value), respectively. P.c, *Phytophthora capsici*; D, days post inoculation.

The expression patterns of the *CaDof*s were differentially regulated in the resistant and susceptible responses against TMV-P0 and TMV-P2, respectively (Fig. 6). *CaDof*32 was highly up-regulated only in the resistant response against TMV-P0, while the expression levels of *CaDof*07, 12, 14, and 15 were increased in the susceptible response against TMV-P2. Some of the *CaDof*s, such as *CaDof*9, 10, 11, 17, and 20, were up-regulated in both responses but showed temporally different expression patterns. Among those, *CaDof*10 showed earlier and higher levels of expression in the resistant response. After PepMoV inoculation, some *CaDof*s were up-regulated, as in the responses to TMV-P0 and TMV-P2, but showed different patterns, such as those of *CaDof*09, 10, 11, 20, and 32 (Fig. 6). *CaDof*04 showed earlier and higher levels of expression after PepMoV inoculation but did not show significant changes after other pathogen inoculations. Other *CaDof*s showed no detectable changes compared with controls and among treatments. In response to *P. capsici* inoculation, nine *CaDof*s were expressed at higher levels compared with the controls (Fig. 6). Among those, *CaDof17* showed higher expression levels compared with those in control and the other viruses treatments. *CaDof*04 was significantly down-regulated. In addition, two genes (*CaDof*05 and 31) showed particularly strong expression levels after *P. capsici* inoculation compared with those after the other treatments. Overall, the expression profiles in response to the biotic stresses suggest diversity and conservation of the biological functions of *CaDof*s during plant-microbe interactions.

## Discussion

The Dofs are a major group of plant-specific TFs involved in diverse functions^4^,^8^. Several studies have addressed the genome-wide identification, function, and evolution of *Dof*s in Arabidopsis, rice^16^, poplar^17^, algae, moss^43^, wheat^18^, sorghum^19^, soybean^20^, Chinese cabbage^22^, and tomato^21^. No previous study has reported the genome-wide identification and transcriptional profiles in response to biotic stresses of *Dof*s in pepper, an agriculturally important member of the Solanaceae. We identified a total of 33 *CaDof*s based on two pepper genomes, *C. annuum* ‘CM334’ and *C. annuum* ‘Zunla-1’. The number of *CaDof*s identified was similar to those in other plants, such as the 36 *AtDof*s in Arabidopsis, the 30 *OsDof*s in rice^16^, and the 34 *SlDof*s in tomato^21^. In addition, the number of reported *Dof*s in many plant species ranges from 20 to 50 genes according to information from the Plant TFDB. Taken together, the numbers of *Dof*s among plant species appear to be regular, regardless of genome size. Consistent with previous results from other plants^16^,^21^, the *CaDof*s were divided into four major groups and seven subgroups based on a neighbor-joining phylogenetic tree. Phylogenetic analysis of the *Dof*s in pepper and tomato revealed particular clusters of paralogous and orthologous gene pairs within each subgroup supported by high bootstrap values (Fig. 1 and Supplementary Fig. S3). Although the clade patterns are consistent with previous results from Arabidopsis, rice, and tomato, the number of duplicated genes within some groups of *Dof*s in pepper were different from those in the other three species. Group B contained the most genes in pepper, whereas group C contains the most genes in Arabidopsis and tomato. The numbers of genes in group A and subgroup D1 in pepper were the same as those in tomato. Previous studies indicated that orthologous *Dof*s in the same clades between monocots and dicots might be derived from common ancestors that evolved before the monocot/dicot split^16^,^21^. Taken together, the results suggest that there were unequal gene duplication events in each clade of the *Dof* family among species, leading to expansion and diversity of the *Dof* repertoire during the course of evolution.

Structural analysis showed that the *CaDof*s contained few introns, with intron numbers ranging from zero to two per gene (Fig. 3). Most of the genes had no introns, and those genes all clustered into the same groups. The lengths of the introns within the genes showed similar patterns within the same groups. Similar characteristics of intron/exon structure were observed in Arabidopsis, rice, and tomato^16^,^21^, suggesting evolutionary conservation of the *Dof*s. The chromosomal locations of the *CaDof*s showed that the *CaDof*s were located on 8 of the 12 pepper chromosomes, with chromosomes 7, 8, 9, and 12 containing no *CaDof*s. Chromosome 2 contained nine *CaDof*s, which was the highest number among the chromosomes. Likewise, chromosomes 7 and 12 in tomato lack *Dof*s, and the highest number of *Dof*s (nine *SlDof*s) in tomato is found on chromosome 2. The chromosomal locations of the orthologous gene pairs between pepper and tomato were compared based on a phylogenetic tree. That comparative analysis revealed that most of the orthologous gene pairs were syntenic between the two plant species. Those results are consistent with previous reports of conserved locations of homologous genes on chromosomes between species^40^,^44^. Hence, we assumed that the locations of the seven unassigned *CaDof*s (*CaDof*27, 28, 29, 30, 31, 32, and 33) might correspond to syntenic positions of orthologous *SlDof*s. For instance, *CaDof*31, which is orthologous to *SlDof*14, might be located on chromosome 3 in pepper.

The motif analysis of *CaDofs* revealed that the *CaDofs* shared conserved motifs in the same subgroup. For example, C1 has specific motifs 7, and 14, whereas C2 contains motif 25. We also found specific motifs in other subgroups: motif 11 and 12 in B1, and motif 3, 4, 5, 8, and 10 in D1 (Fig. 2). The distribution of motifs also showed conserved position of each gene in the same subgroup. These conserved motifs of *Dof* genes indicated that such structures have been preserved by evolution suggesting that these motifs might play important roles in subgroup functions.

Tandem duplication, segmental duplication, and transposition are major evolutionary mechanisms of gene-family expansion^45^. In plants, segmental duplication occurs more frequently than tandem duplication and transposition, because most plants are diploidized polyploids and retain numerous duplicated chromosomal blocks within their genomes^45^. We obtained 10 pairs of paralogs in the pepper *Dof* family based on chromosomal distribution, phylogenetic analysis, and sequence similarity. One pair of paralogs (*CaDof*23 and 24) was the result of a putative tandem duplication event. Among the remaining nine pairs, two pairs (*CaDof*02 and 10 and *CaDof*03 and 09) were preferentially retained duplicates located within a segmental duplication of a single chromosome, whereas seven pairs (*CaDof*01 and 29, *CaDof*12 and 20, *CaDof*13 and 19, *CaDof*15 and 26, *CaDof*17 and 30, *CaDof*21 and 27, and *CaDof*25 and 28) were involved in segmental duplications on different chromosomes. Those results indicate that the *Dof* family in pepper might have evolved mainly via segmental duplications.

We calculated the duplication times of the paralog pairs in the pepper *Dof* family using the Ks value. The monocots/dicot divergence was estimated to occur around 170–235 Mya^46^. The Arabidopsis/tomato and pepper/tomato divergences were estimated to occur around 110–130 Mya and 19.2–20 Mya, respectively^40^,^44^. We estimated that the duplications of nine of the paralogous *Dof* sets in pepper occurred from 194.48 (Ks = 2.37) to 37.06 (Ks = 0.45) Mya and that of one the paralogous gene sets (*CaDof*23 and 24) occurred around 1.45 Mya (Table 2). Therefore, the duplication of most of the *CaDof*s occurred before the pepper/tomato divergence, and only one paralogous pair was duplicated recently, after the pepper/tomato split. Similar cases have been reported in tomato. The duplication of one *SlDof* was estimated to have occurred before the pepper/tomato split (19.1–61.1 Mya)^21^. The Ka/Ks ratio provides a measure of selective pressure. If the gene pair were under purifying selection, it would have been purged by natural selection, presumably because of deleterious effects. Conversely, if the gene pair was under positive selection, it would have been advantageous during the evolution of the two duplicates^47^. We calculated the Ka/Ks ratio of the duplicated pairs in the *CaDof* family. The Ka/Ks ratios of all 10 duplicated pairs in the *CaDof* family were <1.0, suggesting that the duplicated pairs evolved under purifying selection. Duplicate pairs of *Dof*s in tomato and soybeans were also estimated to have evolved under purifying selection^20^,^21^. Overall, the duplication analysis suggests that the segmental expansion of the *CaDof*s might have taken place before the speciation event separating pepper and tomato and that the functional divergence of the *CaDof*s was retained after the duplications.

Transcriptional control of gene expression is an important part of plant growth, development, and response to biotic and abiotic stresses^48^. RNA-seq has been a useful and powerful tool for the analysis of gene expression profiles since next-generation sequencing has become very straightforward. The RNA-seq data showed that most of the *CaDof*s had distinct tissue-specific expression patterns among the five different tissues tested. The *CaDof* expression profiles revealed various patterns among different organs and stages (Fig. 5). *CaDof*17, 18, 21, 27, and 30 were expressed at relatively high levels in various tissues and at several developmental stages. Seventeen of 33 genes showed relatively high expression levels in the root. Similarly, 22 *SlDof*s in tomato and 22 *GmDof*s in soybeans were expressed at relatively high levels in the root^20^,^21^. Ten (*CaDof*08, 11, 13, 17, 18, 21, 22, 27, 29, and 30) and seven (*CaDof*08, 11, 13, 18, 22, 27, and 30) genes showed relatively high expression levels in the stem and leaf, respectively. The expression levels of *CaDof*11, 21, and 30 in the placenta and that of *CaDof*18 in the pericarp were higher than those in other organs, suggesting that those genes could play key roles in fruit development, such as capsaicinoid biosynthesis. Taken together, the results indicate that the *CaDof*s are possibly involved in biological functions during plant development.

Recently, Dof family has also been reported to be possibly involved in biotic stresses^49^,^50^. However, still their role associated with biotic stress were largely unknown. Additional clues and works should be needed to understand and confirm their newer roles in biotic stress. In our study, the *CaDof* family showed distinct expression patterns in response to different biotic stresses (Fig. 6). The transcript levels of the *CaDof*s showed temporal and pathogen-specific variation. *CaDof*04, 05, and 32 showed changes in expression levels in the resistant response to TMV-P0 and/or PepMoV and *P. capsici*, while *CaDof07* was up-regulated only in the susceptible response to TMV-P2. In particular, *CaDof*10 and 11 showed relatively high expression levels in response to each of the viruses, suggesting that they could play a role in defense response against viruses. In addition, some *CaDof*s such as *CaDof*09 and 17, showed similar expression patterns and temporal variation following various pathogen inoculations, indicating a shared function in response to biotic stresses. Thus, the differentially regulated *CaDof*s might play a crucial role in defense against biotic stresses. Those results will provide useful information for the functional characterization of *Dof*s to understand biological processes in pepper and other plants.

## Methods

### Identification and annotation of *CaDof*s

We obtained the consensus amino-acid sequence of the Dof protein as a seed from the Pfam (PF02701) database (http://pfam.sanger.ac.uk/). We then used that sequence to search all of the Dof proteins against the ‘CM334’ genome^40^ using the HMMER software package (Version 3.0, http://hmmer.org/). We confirmed the collected *CaDof*s by a BLAST search against NCBI. We then collected the *CaDof*s manipulated by alignment of the ‘CM334’ genome sequence, the ‘Zunla-1’ genome sequence, the pepper ESTs, and the *Dof* sequences from the Plant TFDB. If the alignment result showed the same sequence among the *Dof*s from several databases, we chose the longest sequence as a representative. We computed the pIs and MWs of the predicted protein sequences using the ExPASy Compute pI/MW tool (http://web.expasy.org/compute_pi/). We obtained the full amino-acid sequences of the tomato *Dof*s, designated *SlDof*s, from the Plant TFDB.

### Motif analysis of the *CaDof*s and exon/intron structural analysis

We used the MEME suite program (http://meme-suite.org/tools/meme) to identify conserved motifs among the *CaDof*s. The analysis settings of the MEME suite were a motif length of 6–200 amino acids and 2–120 motif sites. We set the maximum number of motifs to 25. We set the other conditions to the default values. We performed the alignment of the identified CaDof domains using ClustalW (http://www.ebi.ac.uk/Tools/clustalw/). We predicted the zinc finger structure of the Dof domain based on information from the tomato and Arabidopsis Dof domains^16^,^21^. We searched and confirmed the exon/intron structures of the *CaDof*s by aligning the CDSs with the corresponding genomic regions. We presented the exon/intron structures using the online display tool Gene Structure Display Server (http://gsds.cbi.pku.edu.cn/).

### Phylogenetic tree analysis

We performed a multiple alignment of the 33 full-length CaDofs and 34 full-length SlDofs using ClustalW. We obtained the Dof sequences for tomato from previous research^21^ and the Plant TFDB. We used the alignment result to construct a phylogenetic tree using the neighbor-joining method of MEGA6 software (http://www.megasoftware.net/). We created the phylogenetic tree using the following parameters: Poisson correction, pairwise deletion, and a bootstrap value of 1,000.

### Chromosomal location and calculation of duplication events

We mapped the CDSs of the *Dof*s to the ‘CM334’ chromosomes in the pepper genome database^40^ using BLASTn. Information about the chromosomal locations of the *CaDof*s was based on the identified physical positions on the chromosomes. We used MEGA6 to make the pairwise alignments of paralogs with the removal of gaps. We estimated the Ka, Ks, and Ka/Ks using K-Estimator 6.1v^51^. We then used the Ks value of each pair to estimate the duplication time by following formula: duplication time = Ks/2λ, with the clock-like rate (λ) = 6.1 × 10^−9^ based on previous research^52^.

### Plant materials and pathogen inoculation

We used ‘CM334’ to analyze the responses to TMV-P0, TMV-P2, PepMoV, and *P. capsici*. We conducted the virus inoculations as described by Kang *et al*.^53^. We inoculated *Nicotiana benthamiana* leaves infected with TMV-P0, TMV-P2, and PepMoV mechanically with 0.1 M phosphate buffer (pH 7.0) into true leaves of ‘CM334’ seedlings. For virus inoculation, three independent experiments of each virus were each performed. We performed the preparation and inoculation of *P. capsici* as described by Yeom *et al*.^37^. We inoculated ‘CM334’ seedlings with 1.0 × 10^6^ zoospores/ml *P. capsici*. The pepper plants inoculated with viruses and *P. capsici* were kept in a growth chamber at 23–25 °C under a 16 h/8 h light/dark photoperiod.

### Transcriptome analysis

To obtain RNA-seq data from pathogen-infected pepper tissues, we harvested the leaves from various time points after pathogen inoculation to prepare the total RNA. We used RNA samples extracted from three biological replicates of TMV-P0, TMV-P2, and PepMoV inoculated tissues at various time points for library construction using a modified protocol for strand-specific library construction^54^ (Illumina Inc., San Diego, USA). We sequenced the constructed libraries (insert size: 150–200 bp) using Illumina HiSeq 2000 (Illumina Inc., San Diego, USA). We first aligned the RNA-seq reads to rRNA and tRNA sequences to remove possible contamination. Low-quality reads were filtered by in-house trimming scripts. We then aligned the resulting reads to the reference pepper genome using CLC Assembly Cell (CLC bio, Aarhus, Denmark). Following alignment to each gene model, we normalized the number of mapped reads to the reads per kilobase per million mapped reads (RPKM).

To analyze the expression patterns of the *CaDof*s in specific tissues and at different developmental stages, we used the RNA-seq data obtained from ‘CM334’^40^. We generated transcript data for five tissues: root, stem, leaf, pericarp, and placenta. The pericarp and placenta data included seven developmental stages: 6, 16, and 25 days post inoculation; mature green (MG); breaker (B); and 5 and 10 days post B. We illustrated the log_2_ RPKM values of the expression data for the tissues and developmental stages using the heat-map R package (http://bioconductor.org/). To evaluate the expression patterns in response to the virus and *P. capsici* inoculations, we compared the normalized transcripts obtained under each biotic stress with each negative control (Supplementary Table S2). For data analysis of virus inoculation, three RNA-seq data sets corresponding three biological replicates of each virus were analyzed. Then, mean of values in triplicate datasets were used for analysis of expression pattern and construction of heat maps. We created the heat map using the heat-map R package (http://bioconductor.org/).

## Additional Information

**How to cite this article**: Kang, W.-H. *et al*. Genome-wide analysis of Dof transcription factors reveals functional characteristics during development and response to biotic stresses in pepper. *Sci. Rep.* **6**, 33332; doi: 10.1038/srep33332 (2016).

## Supplementary Material

Supplementary Information

Supplementary table S1

## Figures and Tables

**Table 1:** The *Dof* families and related information of each *Dof* gene in pepper.

Gene name	Gene ID	chromosome or scaffold	ORF length (bp)	No. of aa	pI	MW (kD)
CaDof01	CA01g00490	1	864	287	8.68	32.08
CaDof02	CA02g01120	2	1413	470	5.01	51.57
CaDof03	CA02g01550	2	579	192	9.17	20.82
CaDof04	aCapana02g001770	2	525	174	8.81	19.71
CaDof05	aCapana02g001919	2	924	307	9.71	32.95
CaDof06	CA02g15190	2	1065	354	8.75	38.45
CaDof07	CA02g15750	2	834	277	5.29	31.35
CaDof08	CA02g25910	2	864	287	9.01	31.04
CaDof09	CA02g25980	2	714	237	8.98	25.03
CaDof10	aCapana02g003361	2	1338	445	7.05	48.9
CaDof11	aCapana03g001966	3	801	266	8.55	28.84
CaDof12	CA03g26920	3	969	322	3.61	35.67
CaDof13	CA03g29970	3	1512	503	5.77	54.27
CaDof14	bKS14005F06	4	441	146	8.94	15.8
CaDof15	CA05g08190	5	1410	469	6.28	51.46
CaDof16	CA05g18640	5	702	233	9.79	26.77
CaDof17	CA06g16390	6	618	205	6.57	22.58
CaDof18	CA06g18250	6	933	310	5.47	33.59
CaDof19	CA06g18840	6	1449	482	5.81	52.54
CaDof20	CA06g19670	6	924	307	6.58	33.9
CaDof21	CA06g23590	6	915	304	6.78	33.78
CaDof22	CA06g24550	6	930	309	9.02	33.43
CaDof23	CA10g08680	10	972	323	5.93	36.09
CaDof24	CA10g08690	10	981	326	5.89	36.44
CaDof25	CA10g11420	10	1098	365	9.11	39.69
CaDof26	CA11g04250	11	1308	435	6.54	48.33
CaDof27	CA00g45170	scaffold1106	948	315	6.63	35.38
CaDof28	CA00g57880	scaffold1313	1008	335	9.44	35.76
CaDof29	CA00g64610	scaffold1415	906	301	9.25	32.77
CaDof30	CA00g78480	scaffold1668	642	213	9.12	22.87
CaDof31	CA00g84150	scaffold1807	828	275	4.42	31.06
CaDof32	aCapana12g002748	scaffold1813	1113	370	6.49	40.53
CaDof33	CA00g96300	scaffold4404	648	215	6.62	24

ORF, open reading frame; aa, amino acids; pI, isoelectric point; MW, molecular weight.

^a^Genes are from ‘Zunla-1’ genome.

^b^Genes are from pepper EST.

Others without marks (a or b) in Gene ID panel are from ‘CM334’ genome.

**Table 2:** Duplicated *Dof* members and the dates of the duplication times in pepper.

Gene1	Gene2	aKs	Ka	Ka/Ks	bDate(Mya)
CaDof01	CaDof29	1.17	0.50	0.43	96.30
CaDof02	CaDof10	0.56	0.17	0.29	46.29
CaDof03	CaDof09	1.34	0.40	0.30	109.67
CaDof12	CaDof20	2.37	0.25	0.10	194.48
CaDof13	CaDof19	0.45	0.15	0.34	37.06
CaDof15	CaDof26	0.52	0.10	0.20	42.73
CaDof17	CaDof30	0.95	0.36	0.38	78.24
CaDof21	CaDof27	1.07	0.37	0.35	87.36
CaDof23	CaDof24	0.02	0.01	0.41	1.45
CaDof25	CaDof28	0.96	0.37	0.38	78.89

^a^Ka and Ks were calculated using the program K-Estimator 6.1 v.

^b^The duplication time was estimated according to formula: T = Ks/2λ. The clock like rate (λ) was 6.1 × 10^−9^ subsitutions per site per year^52^. Mya, million years ago.
