# Supplementary material for: Genome-wide analysis of Dof transcription factors reveals functional characteristics during development and response to biotic stresses in pepper
Source: Sci Rep. 2016 Sep 22;6:33332. doi: 10.1038/srep33332 (PMC5032028; doi:10.1038/srep33332)
Supplement: Supplementary Information [file srep33332-s1.pdf]

# Genome-wide analysis of Dof transcription factors reveals functional characteristics during development and response to biotic stresses in peppers

Won-Hee Kang, Seungill Kim, Hyun-Ah Lee, Doil Choi and Seon-In Yeom[1]*

**Supplementary Information**

**Supplementary Table S1;**

**Supplementary Figure S1;**

**Supplementary Figure S2;**

**Supplementary Figure S3;**

Supplementary Table S1. Identified consensus sequence of *CaDof* motifs by MEME

| Motif | Width | Best possible match |
|---|---|---|
| 1 | 57 | EQALKCPRCDSTNTKFCYYNNYNLSQPRHFCKTCRRYWTKGGTL RNVPVGGGCRKNK |
| 2 | 113 | VCLNPTNIDHHNQSTSSSLDHHHGPSIDLALVYSNFLNSTNSKSSQ PEDRQNPELPDD LLLPDQGVLVTPSFELSSMIDMEFVNSELGQESRLLGAGAGDGVD FYFSGIHEEK |
| 3 | 39 | KEKNPERCIWVPKTLRIDDPDEAAKSSIWSTLGIKHDKV |
| 4 | 40 | GGMLFKAFQPKSDERNHEADTSPVLQANPAALSRSLNFQE |
| 5 | 34 | MHHPPFKINGTILAFGSDKPLCESMASVLNIAEK |
| 6 | 30 | WPPPGFPVPFYPAPPYWGCTVAGPWNVPWI |
| 7 | 10 | IHDHHHHEMI |
| 8 | 19 | MEEVRDPAIKLFGKTIQLP |
| 9 | 29 | VMQNCNAFPPHVPCFPGVPWPYPWNAVPW |
| 10 | 15 | PTLGKHSRDEEILKP |
| 11 | 8 | MERGTIWK |
| 12 | 11 | MADRARLAKIP |
| 13 | 21 | HYRHIMISEALQAARVEAPNG |
| 14 | 20 | MGITSLQVCMDSSDWLQDTI |
| 15 | 14 | WPDLAIYTPGSRFK |
| 16 | 38 | DEGSEYEKEDADKDDMTRELNEAKFEEKDQSQMMEESE |
| 17 | 21 | HQLGNNNYMELPPLPCEEIMW |
| 18 | 39 | EPEPEPESAIQNPSHDHSANNANDGSLFNLSNFGNIFKP |
| 19 | 15 | QNVMNHSDVHDDHYT |
| 20 | 11 | DFMESKYEALV |
| 21 | 14 | MMTTVKQEMCGMAR |
| 22 | 37 | TADESVEPEISSGISDDPKMQDAEKEILSPKSIEEED |
| 23 | 17 | MDVKPNPKILSLEWHDQ |
| 24 | 9 | KVLWGFPWQ |
| 25 | 10 | YWNGMLGGGG |

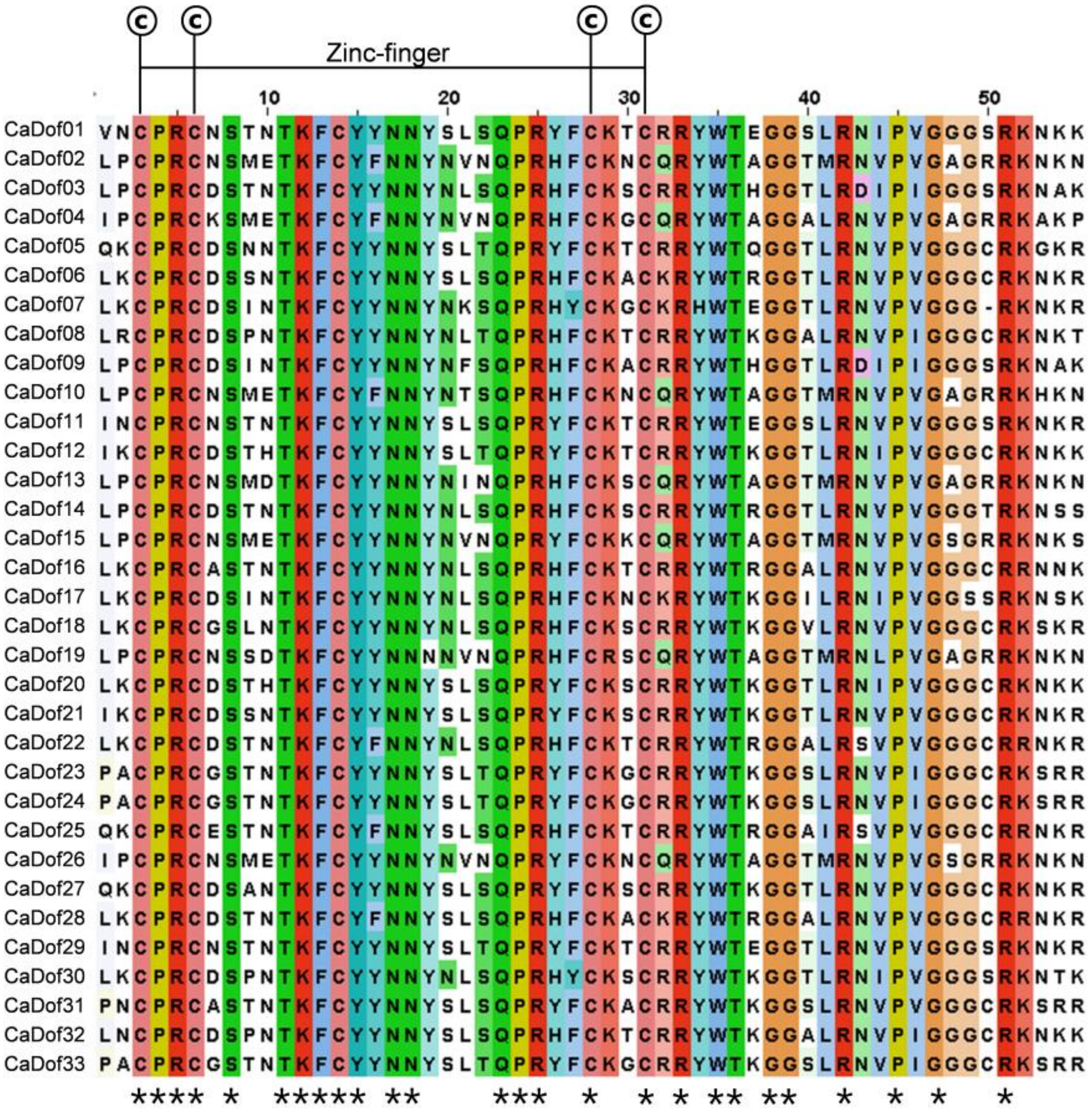


Supplementary Figure S1. Multiple sequence alignment of Dof domain from the identified *CaDof* genes in pepper. The four cysteine residues for zinc finger structure are completely conserved. Completely conserved residues are indicated bottom line of alignment with asterisks. The numbers above amino acids indicate the position of each amino acid of Dof domain.

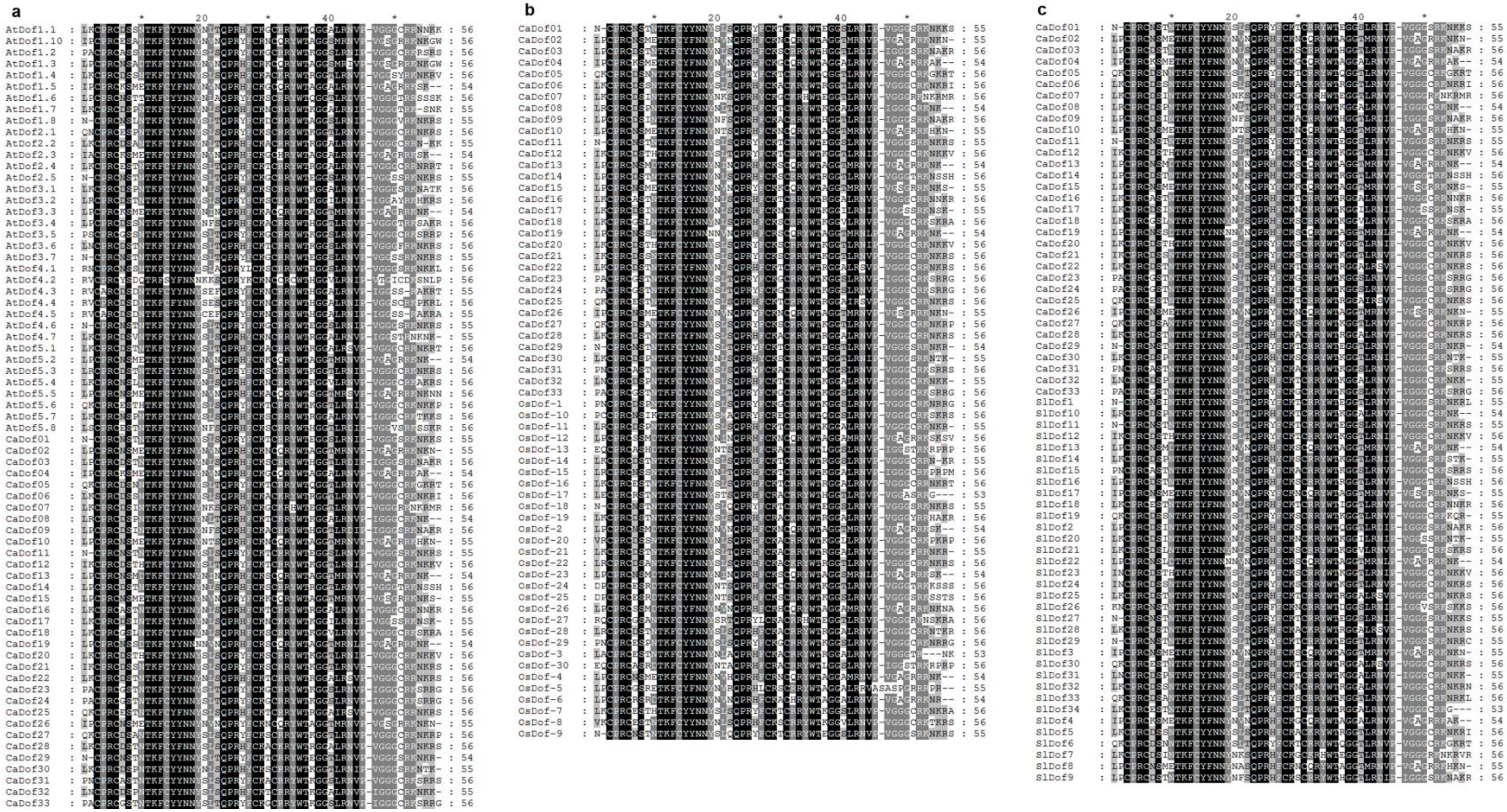

Supplementary Figure S2. Multiple sequence alignment of Dof domain in pepper, Arabidopsis, rice and tomato. Full length sequence of Dof proteins in pepper were aligned with that of Arabidopsis (a), rice (b), and tomato (c), respectively. The highly conserved sequences (Dof domain residues) in four plants were represented in supplementary figure 1. Identical residues in alignment were shaded by black color. The numbers above amino acids indicate the position of each amino acid of Dof domain.

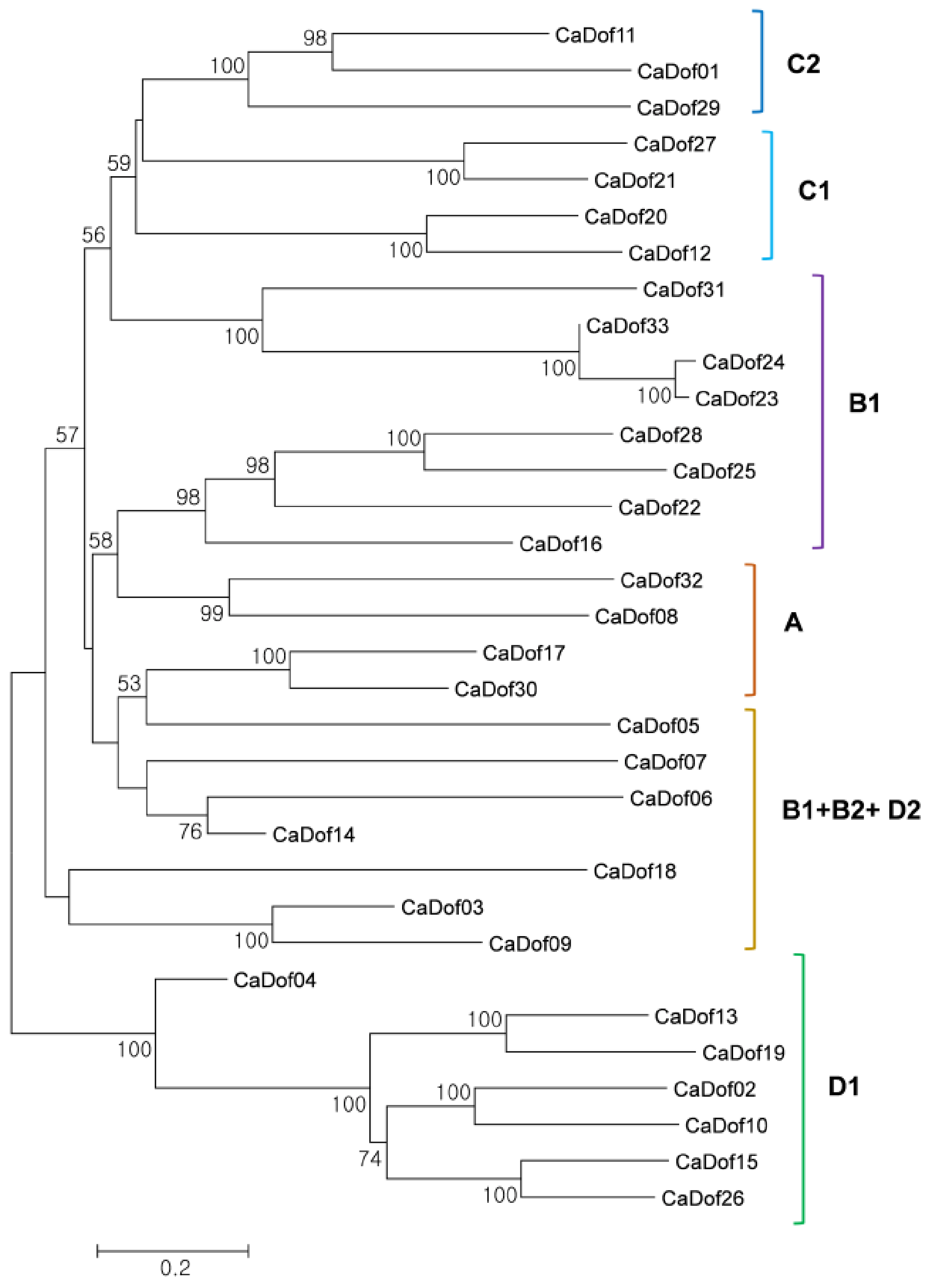


Supplementary Figure S3. Phylogenetic analysis of *Dof* genes in pepper (*C. annuum*). The tree was constructed by the neighbor-Joining method with 1,000 bootstrap replicates after the alignment of 33 Dof proteins using Clustal W.
